# Commentary: Alpha Synchrony and the Neurofeedback Control of Spatial Attention

**DOI:** 10.3389/fnins.2020.00597

**Published:** 2020-06-16

**Authors:** Christopher Gundlach, Norman Forschack

**Affiliations:** ^1^Experimental Psychology and Methods, Universität Leipzig, Leipzig, Germany; ^2^Department of Neurology, Max Planck Institute for Human Cognitive and Brain Sciences, Leipzig, Germany

**Keywords:** vision, alpha-rhythm, neurofeedback, spatial attention, EEG

Alpha-band activity is one neural signature, long speculated to be involved in biasing neural processing toward attended information (see Van Diepen et al., 2019). Many studies propose alpha-lateralization, i.e., a concomitant decrease in alpha-band power in one hemisphere and an increase in the other, as a neural marker of shifts in visuospatial attention. In recent work, Bagherzadeh et al. (2019) examined the potential causal role of alpha-band modulations for the deployment of visuospatial attention. In a neurofeedback task, participants learned to upregulate parietal alpha-band amplitude-lateralization, while markers of attentional shifting were measured. Crucially, enhanced alpha-lateralization at left and right parietal MEG sensors was beneficial for doing well in the orientation match-to-sample task because it increased the contrast of the to-be-remembered stimulus. The central question was whether upregulated alpha-lateralization led to a corresponding shift in visuospatial attention. Across different measures, evidence was provided for such a shift: (1) For the neurofeedback task, the authors reported enhanced probe-related evoked responses contralateral to the hemisphere, for which alpha was downregulated. Outlasting the neurofeedback task, (2) alpha-band power, and (3) reaction times still depicted lateralization for neutral trials of a subsequent Posner-paradigm. Finally, (4) gaze orientation shifted contralaterally to the hemisphere showing decreased alpha in a free-viewing task.

These measures led the authors to conclude that the increased alpha-lateralization during neurofeedback *caused* a shift in spatial attention (see Figure 1A). To claim that alpha-lateralization causes attentional shifts the reverse, however, i.e., a strategy that deploys covert spatial attention in order to increase alpha-lateralization, has to be ruled out. In our notion, there are some caveats in the author's line of argumentation, and the data provides indeed some evidence that subjects used spatial attention (by attending to a lateralized aspect of the central stimulus) to alter their alpha-lateralization (Figure 1B).

**Figure 1:**
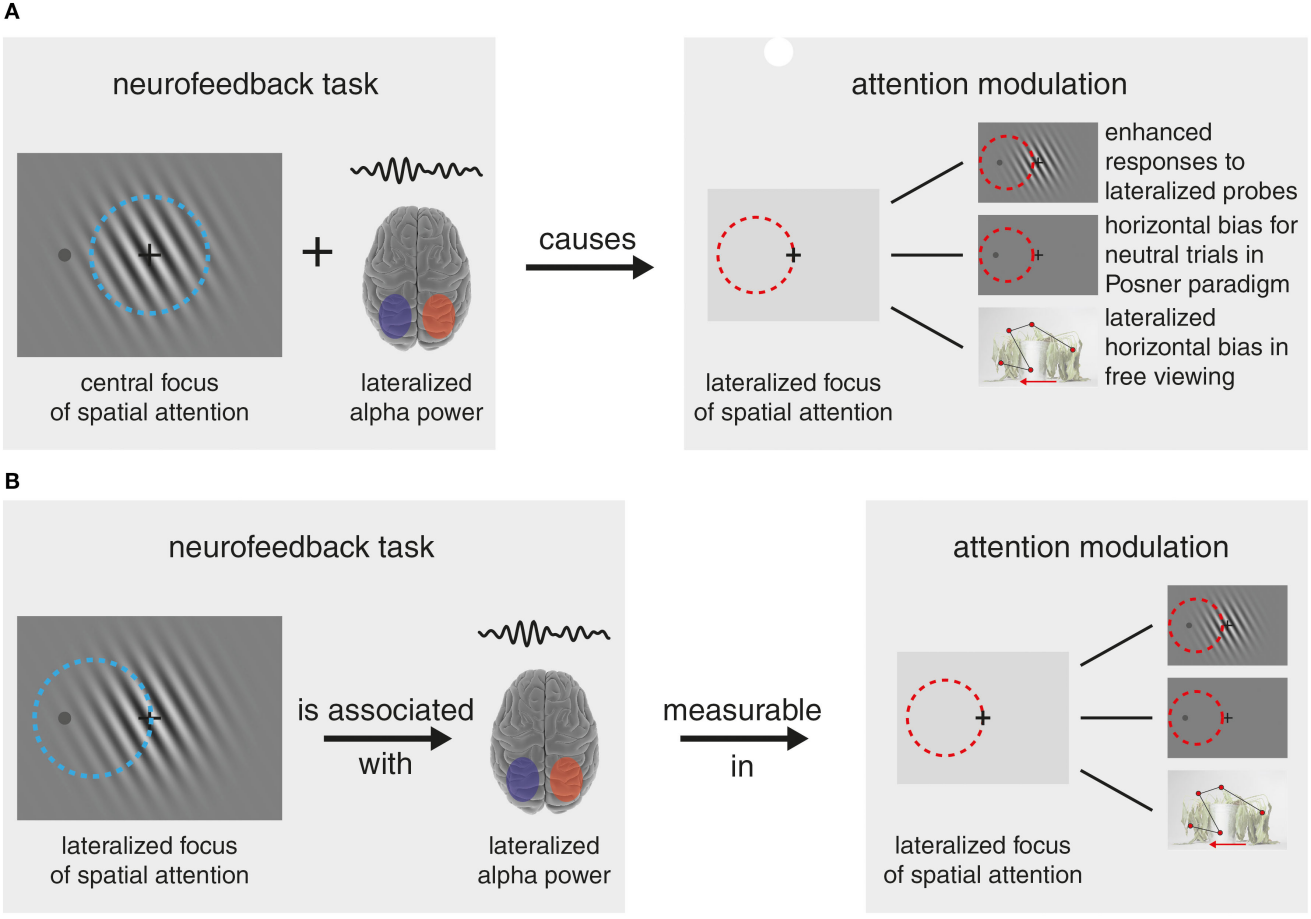
Lines of Argumentation **(A)** The authors' line of argumentation sees the training of parietal alpha-band amplitude lateralization (in the example left > right) in the neurofeedback task independent from a central focus of spatial attention. The trained alpha-lateralization then caused a corresponding prolonged bias/shift in the focus of spatial attention (here to the left), which manifested in enhanced responses to lateralized probes during the neurofeedback task, an outlasting horizontal bias in neutral trials of a Posner paradigm task and a lateralized bias in a free-viewing task. **(B)** The alternative reasoning that explains the main experimental findings equally well: In order to lateralize individual alpha-band power, participants covertly shifted spatial attention toward a lateral portion of the centrally presented stimulus. Note, claiming a causal relationship between alpha-lateralization and spatial attention, is not warranted in the presence of competing explanations. Note that some figure content is adapted from https://www.somersault1824.com/ under CC BY-NC-SA 4.0 license.

The authors stated that it was unclear which strategies participants used to lateralize the alpha-amplitude and proposed that shifting attention *per se* was not required for the task because it only involved a centrally presented stimulus and that participants thus relied on contingent feedback to learn to alter alpha-lateralization. Nonetheless, covertly shifting attention represents an effective strategy to generate reliable modulation of measurable alpha-band activity often exploited in BCIs (Jensen et al., 2011; Treder et al., 2011).

To control that subjects indeed refrained from using a spatial-attention-related strategy, the authors compared the direction of microsaccades as a marker of covert spatial attention (Engbert and Kliegl, 2003; Lowet et al., 2018) between the neurofeedback task and a follow-up Posner-paradigm. They found a bias in microsaccades toward the cued side during the Posner-paradigm but not during the neurofeedback task. The authors interpreted this as evidence for a *non*-spatial-attention-related strategy during the neurofeedback task. Although the analysis of potentially biased microsaccades seems to be a valid marker of spatial attentional deployment, there may be some alternative explanations and challenges to the employed analysis and the interpretation of the findings at hand. First, the interpretation of the null-effects may be difficult because null-effects in conventional significance testing do not provide evidence for the null-hypothesis (in contrast to Bayesian analysis approaches, Rouder et al., 2009). Furthermore, the comparison of microsaccade-related measures between two physically different tasks (neurofeedback vs. Posner-paradigm) may be challenging. First, a presumed attentional shift during the neurofeedback task (see Figure 1B, ≈ 3.4°) would not necessarily be as large as the required shift for the more eccentric stimuli in the Posner-paradigm (6.7°), which may affect the strength of measurable microsaccades (Casteau and Smith, 2018). For the Posner-paradigm, this shift is in the range usually reported in studies on microsaccades and attention (≥4°) (Engbert and Kliegl, 2003; Yuval-Greenberg et al., 2014), but the shift required during the neurofeedback task would be well below this typical range. Second, previous studies usually examined an attentional shift *toward* an object at a different position but not toward an aspect/position *within* the same object. As neural processes relevant for object-based and spatial attention may differ (Chen, 2012), they may also bias microsaccades differently. Third, the size of microsaccades differs depending on the spatial frequency of objects presented in the background (Amit et al., 2019), again rendering comparisons between both tasks difficult (if not impossible). Thus, the only *direct* evidence against a spatial-attention-related strategy during the neurofeedback task put forward by the authors may suggest alternative interpretations of the data and is thus not entirely convincing.

Furthermore, the results of the analysis of microsaccadic shifts are incoherent with respect to other measures of attention rendering an unequivocal interpretation difficult. While non-biased microsaccades in the neurofeedback task are interpreted as evidence for the absence of a spatial-attentional shift, such a bias was also absent for the *neutral* Posner trials. Nevertheless, for this condition, a bias in attention was proclaimed because alpha-power and reaction times were lateralized according to the trained direction in the neurofeedback task.

Most crucially, for the neurofeedback task there is a finding strongly pointing toward employed spatial attention: Responses to probes, laterally presented during this task, show a clear amplification for the trained side. Such amplification is usually interpreted as a sensory gain control-related modulation, associated with a shift of spatial attention (Hillyard et al., 1998). Confusingly, this finding is described as a “bias in visual processing,” which is probably the most pointed description of an effect of attention, but not interpreted as related to attention. Thus, ultimately the findings seem more concordant with an idea that participants used a covert-spatial-attention-related strategy to alter the alpha-lateralization (see Figure 1B). Hence, the claim alpha-lateralization causes a shift in spatial attention cannot be made unequivocally.

Methodologically, Bagherzadeh et al. (2019) reported a state-of-the-art study tackling a very relevant and pressing research question of how selective attention is implemented in the human brain. By approaching such research questions from different and innovative methodological angles, as done in the discussed study, future work will much more likely elucidate, for instance, the intertwined relationship between alpha-lateralization and attention. The modulation of alpha lateralization and evoked potentials combined with readily available neurotechnology will certainly propel knowledge accumulation in the near future and impact applications beyond basic science, e.g., in neuropathology. However, beyond all methodological efforts, we want to stress that a claim, as far stretching as “alpha is causally involved in modulating spatial attention” (for a controversial view see Tune et al., 2019; Gundlach et al., 2020), any confound between alpha-lateralization and spatial attention during the neurofeedback task needs to be ruled out or controlled. This could mean to examine or experimentally manipulate different strategies during the neurofeedback task to simplify the identification of causal structures (Grosse-Wentrup et al., 2016) and control for reverse causation (Angrist and Pischke, 2008; Spirtes and Zhang, 2016). Furthermore, it is important to go beyond linear correlation tests that might occlude potential non-linear dependencies in neuroscientific data (Grosse-Wentrup et al., 2011; Forschack et al., 2017). All these approaches will (hopefully) help to represent the potentially complex relationship between attention and neural activity on a level that is neither too simplified nor too complex.

## Author Contributions

All authors listed have made a substantial, direct and intellectual contribution to the work, and approved it for publication.

## Conflict of Interest

The authors declare that the research was conducted in the absence of any commercial or financial relationships that could be construed as a potential conflict of interest.

## References

[B1] Amit R. Abeles D. Yuval-Greenberg S. (2019). Transient and sustained effects of stimulus properties on the generation of microsaccades. J. Vis. 19:6. 10.1167/19.1.6 30640374

[B2] Angrist J. D. Pischke J.-S. (2008). Mostly Harmless Econometrics: An Empiricist's Companion. Princeton, NJ: Princeton University Press.

[B3] Bagherzadeh Y. Baldauf D. Pantazis D. Desimone R. (2019). Alpha synchrony and the neurofeedback control of spatial attention. Neuron 105, 577–587.e5. 10.1016/j.neuron.2019.11.001 31812515

[B4] Casteau S. Smith D. T. (2018). Covert attention beyond the range of eye-movements: evidence for a dissociation between exogenous and endogenous orienting. Cortex 122, 170–186. 10.1016/j.cortex.2018.11.007 30528427

[B5] Chen Z. (2012). Object-based attention: a tutorial review. Atten. Percept. Psychophys. 74, 784–802. 10.3758/s13414-012-0322-z 22673856

[B6] Engbert R. Kliegl R. (2003). Microsaccades uncover the orientation of covert attention. Vision Res. 43, 1035–1045. 10.1016/S0042-6989(03)00084-1 12676246

[B7] Forschack N. Nierhaus T. Müller M. M. Villringer A. (2017). Alpha-band brain oscillations shape the processing of perceptible as well as imperceptible somatosensory stimuli during selective attention. J. Neurosci. 37, 6983–6994. 10.1523/JNEUROSCI.2582-16.2017 28630252 PMC6705724

[B8] Grosse-Wentrup M. Janzing D. Siegel M. Schölkopf B. (2016). Identification of causal relations in neuroimaging data with latent confounders: an instrumental variable approach. Neuroimage 125, 825–833. 10.1016/j.neuroimage.2015.10.062 26518633

[B9] Grosse-Wentrup M. Schölkopf B. Hill J. (2011). Causal influence of gamma oscillations on the sensorimotor rhythm. Neuroimage 56, 837–842. 10.1016/j.neuroimage.2010.04.265 20451626

[B10] Gundlach C. Moratti S. Forschack N. Müller M. M. (2020). Spatial attentional selection modulates early visual stimulus processing independently of visual alpha modulations. Cereb. Cortex 30, 3686–3703. 10.1093/cercor/bhz335 31907512

[B11] Hillyard S. A. Vogel E. K. Luck S. J. (1998). Sensory gain control (amplification) as a mechanism of selective attention: electrophysiological and neuroimaging evidence. Philos. Trans. R. Soc. Lond. B Biol. Sci. 353, 1257–1270. 10.1098/rstb.1998.0281 9770220 PMC1692341

[B12] Jensen O. Bahramisharif A. Oostenveld R. Klanke S. Hadjipapas A. Okazaki Y. O.. (2011). Using brain–computer interfaces and brain-state dependent stimulation as tools in cognitive neuroscience. Front. Psychol. 2:100. 10.3389/fpsyg.2011.00100 21687463 PMC3108578

[B13] Lowet E. Gomes B. Srinivasan K. Zhou H. Schafer R. J. Desimone R. (2018). Enhanced neural processing by covert attention only during microsaccades directed toward the attended stimulus. Neuron 99, 207–214.e3. 10.1016/j.neuron.2018.05.041 29937279 PMC8415255

[B14] Rouder J. N. Speckman P. L. Sun D. Morey R. D. Iverson G. (2009). Bayesian t tests for accepting and rejecting the null hypothesis. Psychon. Bull. Rev. 16, 225–237. 10.3758/PBR.16.2.225 19293088

[B15] Spirtes P. Zhang K. (2016). Causal discovery and inference: concepts and recent methodological advances. Appl. Inform. 3:3. 10.1186/s40535-016-0018-x 27195202 PMC4841209

[B16] Treder M. S. Bahramisharif A. Schmidt N. M. van Gerven M. A. J. Blankertz B. (2011). Brain-computer interfacing using modulations of alpha activity induced by covert shifts of attention. J. Neuroeng. Rehabil. 8, 24. 10.1186/1743-0003-8-24 21672270 PMC3114715

[B17] Tune S. Fiedler L. Alavash M. Obleser J. (2019). Individual listening success explained by synergistic interaction of two distinct neural filters. bioRxiv [Preprint]. 10.1101/512251

[B18] Van Diepen R. Foxe J. J. Mazaheri A. (2019). The functional role of alpha-band activity in attentional processing: the current zeitgeist and future outlook. Curr. Opin. Psychol. 29, 229–238. 10.1016/j.copsyc.2019.03.015 31100655

[B19] Yuval-Greenberg S. Merriam E. P. Heeger D. J. (2014). Spontaneous microsaccades reflect shifts in covert attention. J. Neurosci. 34, 13693–13700. 10.1523/JNEUROSCI.0582-14.2014 25297096 PMC4188967

